# Profiling microRNAs in individuals at risk of progression to rheumatoid arthritis

**DOI:** 10.1186/s13075-017-1492-9

**Published:** 2017-12-22

**Authors:** L. Ouboussad, L. Hunt, E. M. A. Hensor, J. L. Nam, N. A. Barnes, P. Emery, M. F. McDermott, M. H. Buch

**Affiliations:** 1 Leeds Institute of Rheumatic and Musculoskeletal Medicine (LIRMM), University of Leeds, Chapel Allerton Hospital, Chapeltown Road, Leeds, LS7 4SA UK; 2 National Institute for Health Research - Leeds Biomedical Research Centre (NIHR-LBRC), Leeds Teaching Hospitals NHS Trust, Leeds, UK; 3 Present Address: Faculty of Life Sciences, University of Manchester, Manchester, UK

**Keywords:** Rheumatoid arthritis, MicroRNA, At risk, Progression, ACPA, Early RA

## Abstract

**Background:**

Individuals at risk of rheumatoid arthritis (RA) demonstrate systemic autoimmunity in the form of anti-citrullinated peptide antibodies (ACPA). MicroRNAs (miRNAs) are implicated in established RA. This study aimed to (1) compare miRNA expression between healthy individuals and those at risk of and those that develop RA, (2) evaluate the change in expression of miRNA from “at-risk” to early RA and (3) explore whether these miRNAs could inform a signature predictive of progression from “at-risk” to RA.

**Methods:**

We performed global profiling of 754 miRNAs per patient on a matched serum sample cohort of 12 anti-cyclic citrullinated peptide (CCP) + “at-risk” individuals that progressed to RA. Each individual had a serum sample from baseline and at time of detection of synovitis, forming the matched element. Healthy controls were also studied. miRNAs with a fold difference/fold change of four in expression level met our primary criterion for selection as candidate miRNAs. Validation of the miRNAs of interest was conducted using custom miRNA array cards on matched samples (baseline and follow up) in 24 CCP+ individuals; 12 RA progressors and 12 RA non-progressors.

**Results:**

We report on the first study to use matched serum samples and a comprehensive miRNA array approach to identify in particular, three miRNAs (miR-22, miR-486-3p, and miR-382) associated with progression from systemic autoimmunity to RA inflammation. MiR-22 demonstrated significant fold difference between progressors and non-progressors indicating a potential biomarker role for at-risk individuals.

**Conclusions:**

This first study using a cohort with matched serum samples provides important mechanistic insights in the transition from systemic autoimmunity to inflammatory disease for future investigation, and with further evaluation, might also serve as a predictive biomarker.

**Electronic supplementary material:**

The online version of this article (doi:10.1186/s13075-017-1492-9) contains supplementary material, which is available to authorized users.

## Background

Individuals at risk of rheumatoid arthritis (RA) or preclinical RA [1, 2] are characterised by the presence of systemic autoimmunity in the form of highly specific anti-citrullinated peptide antibodies (ACPAs) with or without rheumatoid factor (RF) [3]. Increasing research efforts are focusing on tools to identify those at highest risk of progression to RA in whom immunomodulatory therapy could be used as a preventative strategy [4]. We have previously reported on an at-risk cohort defined by the presence of anti-cyclic citrullinated peptide (CCP) antibody and non-specific musculoskeletal (MSK) symptoms [5] in whom progression to inflammatory arthritis (IA) occurred in 50%, after a median of 7.9 months (range 0.1–52.4), 34% within 12 months.

MicroRNAs (miRNAs) are a highly conserved class of short non-coding RNAs that serve as transcriptional negative regulators [6]. A number of studies have demonstrated dysregulated miRNA expression within the inflamed joints of patients with RA, with in particular, evidence for miR-146a and miR-155 upregulation [7, 8]. Such studies to date, however, have focused on the established phase of RA. Arguably, alternative additional miRNAs might be relevant in driving a state of autoimmunity to disease i.e. at the time of disease initiation. No miRNA study to date has focused on individuals at risk of RA.

We took a unique approach of using samples from individuals identified as being at risk and a further sample at the time of their development to RA; first, to compare miRNA expression between health and individuals at risk of RA, and those that develop RA; second, to evaluate for any change in expression of the identified miRNAs with progression along the RA continuum and third, to explore whether these miRNAs could inform a signature predictive of progression from systemic autoimmunity to RA for future evaluation.

## Methods

### Study design and participants

#### Prospective CCP cohort study

Since 2007, patients ≥ 18 years of age presenting to primary care services in Yorkshire (UK) with any new, non-specific, MSK symptom(s), who test positive for anti-CCP (CCP+) have been invited to attend regular assessments at the research clinic at Chapel Allerton Hospital, Leeds (UK), as part of the prospective CCP study. The CCP study is sponsored by the University of Leeds, approved by the appropriate research ethics committee (REC; reference 06/Q1205/169). All patients provided written informed consent for the study.

#### Patient assessments

All patients recruited to the CCP study are assessed at baseline, 3-monthly intervals for the first year, and then as clinically indicated and/or until they developed inflammatory arthritis (IA); defined by the presence of at least one tender and swollen joint confirmed by a rheumatologist. Rheumatologists, trained in the assessment of IA, carry out the clinical assessments. Blood sampling and power Doppler ultrasound scan of the hands and feet are performed at baseline and then at regular intervals until the development of IA. The ultrasound examination was performed on all patients by a single rheumatologist (JLN) experienced in MSK ultrasound. Patients recruited to the study had scans of the wrists, metacarpophalangeal joints (MCPs), proximal interphalangeal joints (PIPs) and metatarsophalangeal joints (MTPs) bilaterally (as well as any other joints if symptomatic). We and others have previously demonstrated the presence of synovitis on ultrasound [9]. CCP+ at-risk individuals with ultrasound-identified synovitis (defined as power Doppler signal) of the aforementioned small joints were excluded, as this was considered to be too close to RA on a pathological level. Selection of individuals for our miRNA profiling study was from this at-risk, CCP+ cohort.

### Pilot and validation-phase patient cohorts

From our at-risk cohort, 12 CCP+ patients who progressed to RA (American College of Rheumatology (ACR)/European League Against Rheumatism (EULAR) 2010 criteria), termed very early RA (VERA) were available as per the criteria described earlier and selected. Each patient had a blood sample taken at baseline and at the time of detection of synovitis, forming the matched element of this analysis. Twelve healthy controls (HC) were identified via our “ask a friend” approach, tested for and confirmed to be anti-CCP negative, and subsequently also included.

To validate the findings, a further 12 CCP+ patients who progressed to VERA (progressors) and had a matched blood sample available at detection of synovitis were identified. A comparator group consisting of the available 12 CCP+ individuals who did not progress to VERA (non-progressors) and for whom samples were available, were also selected, with a matched sample used 36 weeks after baseline. The 36-week time point was selected following pilot-phase data, indicating a median time to progression to synovitis of 34.5 weeks, enabling a closely matched sample point between the two groups. A further 12 HC were studied. Thus, the validation phase included an identical group to that in the pilot phase and an additional comparator group to enable us to determine whether the miRNAs identified were unique to the development of VERA. Additional file 1 illustrates patient characteristics for the pilot and validation phases.

#### Isolation and profiling of serum miRNA

Serum microRNAs were isolated according to the manufacturer’s protocol using miRNeasy serum plasma kit (Qiagen, UK). For complementary DNA (cDNA) synthesis, Taqman miRNA reverse transcription kit was used (Life Technologies), 3 μl RNA input isolated from serum with Megaplex primer pools Human set v3.0 A and B (Life Technologies) separately. Pre-amplification reactions were performed following manufacturer’s protocol using Taqman pre-amplification mastermix, Taqman array human miRNA A and B (Life Technologies). Undiluted pre-amplification product was prepared in a mastermix with Taqman universal mastermix II no UNG and water and loaded into Taqman Low Density TLDA microRNA cards A v3.0 and B set v2.0 (Life Technologies) on an Applied Biosystems 7900HT fast real-time system. See Additional file 2 for detailed methods.

#### Quantitative reverse transcription (qRT)-PCR on custom miRNA cards

The validation phase was carried out using quantification of the expression of miRNAs of interest using TLDA custom cards (31 candidate miRNAs), and RNU6B was used as control for normalisation as recommended by the manufacturer. Expression profiles of RNU6B were stable across all samples with cycle threshold (Ct) values ranging between 23 and 26. Extraction of serum RNA was as described above (detailed method in Additional file 2). Custom primers for the selected miRNAs were used for reverse transcription and pre-amplification steps. Expression of each miRNA and control was measured in triplicates, and four samples could be included on each card; baseline and follow-up samples for pairs of patients were therefore assigned to the cards. For each individual sample the mean of the three endogenous control replicates was used to normalise values for each of the three replicates per miRNA, then the mean delta Ct (dCt) per miRNA was taken.

### miRNAs network analysis

MetaCore™, an extensively used integrated software suite (Thompson Reuters, New York, NY, USA) used for the functional analysis of high-throughput data including microRNA, and based on MetaBase [10, 11] was used for the network analysis.

### Statistical analysis

For both phases, we used a rule of thumb of n = 12 per group for pilot studies [12]. As appropriate for pilot studies, the extent of descriptive differences rather than inferential testing (and use of *p* values) was applied. For between-group comparisons, quantile regression, adjusting for age, was used to obtain adjusted between-group differences in median dCt, which was converted to fold difference (FD) (2^-ddCt^). For within-patient changes, ddCt was calculated then median ddCt was calculated at the group level and converted to fold change (FC). If FD or FC was < 1, -1/(value) was calculated. Fold differences were calculated as 2-(dCt (progressors)-dCt (non-progressors)). Fold changes were calculated as 2-(dCt (follow-up)-dCt (baseline)). In either case, if the value was < 1, it was transformed to -1/FD (or -1/FC as appropriate). Negative values therefore indicate that expression was lower in progressors compared to non-progressors (negative FD), or lower at follow up compared to baseline (negative FC).

To identify the most dysregulated miRNAs to take into the validation phase, particularly stringent criteria were applied of |FD| or |FC| ≥ 4, irrespective of statistical significance, and within progressors, we additionally required the direction of change to be consistent in ≥ 75% of patients. Association with clinical variables was assessed using Spearman’s rank. Area under the receiver operating characteristic (ROC) curve for classifying progressors/non-progressors was calculated for each miRNA. Sensitivity/specificity was calculated at the point that maximised the Youden index (sensitivity + specificity-1). In the validation phase undetermined Ct values were imputed prior to analysis (see Additional file 2). GraphPad Prism 5, R and SPSS v.21 software packages were used.

## Results

### Patient cohorts - progression from CCP+ status to VERA

CCP+ patients (n = 136) with non-specific MSK symptoms were recruited to the prospective “at-risk” clinic: 57 patients progressed to VERA after a median (range) of 8.6 months (0.1–52.4). Of those 57 patients, 29 had no ultrasound-detectable synovitis (including in symptomatic joints) at baseline; of these, 12 available individuals were selected for the pilot phase. A further available 24 patients (12 who progressed to RA and 12 who did not) were selected for the validation phase (Additional file 1).

### Pilot phase of serum miRNA profiling

Of the 754 human miRNAs accurately quantified, a number were observed to have different expression profiles between the cohorts. As detailed earlier, the primary criterion for selection of miRNAs of interest was a FD/FC of 4 in expression level (FD/FC ≥4); for within-patient change (CCP+ status to VERA) we also required a pattern of dysregulation consistent across ≥ 75% of the cohort.

A list of miRNAs of interest was established (Table 1) comprising all dysregulated miRNAs across the three studied cohorts (19 in total), 9 miRNAs (miR-21, miR-146a, miR-155, miR-18a, miR-34a, miR-203, miR-223, miR-16, miR-132) that have been demonstrated to be involved in the pathogenesis in RA from the literature [13] and 2 miRNAs (miR-15#, miR-335#) that started to be expressed as patients progressed from CCP to VERA. Following adjustment for age, 2 of these miRNAs (miR-374 and miR-454) no longer had an FD >4 but were still included in the final 31 miRNAs of interest.

**Table 1:** List of miRNAs of interest with age-adjusted FD ≥4 between the three studied cohorts in the pilot phase (upregulated FC ≥4, downregulated FC ≤ -4)

	HC	CCP	VERA	CCP vs. HC	VERA vs. HC	CCP to VERA (within progressors)		
miR	dCt median (IQR)	dCt median (IQR)	dCt median (IQR)	FD between medians	FD between medians	Median (IQR) ddCt	Median FC	Number upregulated (/12)
miR-16	-6.3 (-7.1, -6.0)	-7.1 (-7.6, -7.0)	-7.6 (-8.2, -7.4)	1.7	2.4	-0.4 (-1.1, -0.1)	1.3	10
miR-18a	0.1 (-0.8, 1.3)	1.3 (0.3, 2.0)	-0.1 (-1.0, 0.4)	-2.4	1.1	-1.6 (-2.0, -1.0)	3.1	10
miR-19a	-0.9 (-1.6, 1.0)	-2.4 (-2.8, -1.9)	-3.2 (-3.5, -2.8)	2.9	5.0	-0.6 (-1.7, 0.1)	1.5	9
miR-21	-2.8 (-4.1, -2.5)	-3.8 (-4.4, -3.3)	-4.3 (-4.8, -4.1)	2.0	2.7	-0.7 (-1.2, -0.2)	1.6	9
miR-22	4.2 (0.5, 4.8)	3.0 (1.5, 5.1)	0.9 (0.6, 1.5)	2.2	9.4	-2.1 (-3.6, -1.5)	**4.3**	**12**
miR-26b	-1.4 (-3.0, 0.2)	-3.1 (-3.4, -1.8)	-3.6 (-4.2, -2.8)	3.3	4.7	-0.7 (-2.3, -0.3)	1.7	10
miR-34a	-0.2 (-2.1, 0.8)	-0.1 (-0.3, 1.0)	-0.6 (-2.1, 0.2)	-1.1	1.3	-0.1 (-0.9, 0.3)	1.1	6
miR-101	2.5 (1.7, 3.2)	1.6 (1.3, 1.8)	0.4 (-0.3, 0.9)	1.9	4.3	-1.1 (-1.9, -0.6)	2.1	11
miR-132	-1.6 (-2.0, -1.4)	-1.7 (-2.1, -1.6)	-2.5 (-2.7, -2.2)	1.0	1.8	-0.8 (-1.1, -0.3)	1.7	11
miR-142-3p	-2.3 (-4.5, -1.3)	-4.4 (-4.5, -3.8)	-5.0 (-5.2, -4.5)	4.2	6.2	-0.4 (-1.1, -0.3)	1.4	10
miR-142-5p	3.9 (2.9, 5.7)	3.0 (2.4, 5.5)	1.7 (1.5, 4.3)	1.9	4.7	-1.3 (-1.4, -0.3)	2.4	10
miR-146a	-7.3 (-7.5, -6.5)	-7.1 (-7.6, -7.0)	-7.5 (-7.7, -7.3)	-1.2	1.1	-0.5 (-0.8, 0.2)	1.4	8
miR-155	-0.9 (-2.3, 1.5)	0.1 (-0.5, 1.1)	-0.5 (-0.7, -0.3)	-2.1	-1.4	-0.3 (-1.3, 0.1)	1.2	8
miR-195	-2.5 (-3.0, -1.6)	-0.4 (-3.4, 0.2)	-2.9 (-4.2, -0.5)	-4.6	1.3	-1.1 (-2.0, -0.5)	2.1	11
miR-197	-3.5 (-3.3, -1.3)	0.6 (-1.5, 2.9)	0.6 (-2.5, 2.2)	-16.8	-16.5	-0.6 (-2.7, 1.3)	1.5	7
miR-203	2.3 (1.8, 3.0)	3.0 (2.1, 3.0)	2.9 (2.6, 3.4)	-1.5	-1.5	0.0 (-0.4, 0.6)	1.0	6
miR-210	3.9 (0.1, 5.0)	3.1 (2.2, 3.4)	1.5 (1.1, 1.8)	1.8	5.1	-1.8 (-2.9, -0.3)	3.4	10
miR-223	-9.3 (-9.6, -8.8)	-9.9 (-9.9, -9.6)	-10.3 (-10.4, -10.0)	1.5	2.0	-0.4 (-0.6, 0.2)	1.3	6/9^a^
miR-361	-0.1 (-1.1, 1.1)	2.1 (-0.5, 2.5)	0.6 (-0.2, 0.7)	-4.7	-1.7	-1.5 (-2.1, -1.0)	2.9	11
miR-374^d^	-2.2 (-3.4, -2.1)	-3.2 (-3.7, -2.0)	-4.1 (-4.3, -3.9)	2.0	3.8	-0.6 (-1.0, -0.4)	1.5	12
miR-382	-0.7 (-1.2, 0.5)	1.4 (0.9, 2.7)	-0.1 (-1.2, 0.5)	-4.1	-1.5	-2.0 (-2.8, -0.8)	**4.1**	**11**
miR-454^d^	-1.3 (-2.8, 0.2)	-2.3 (-3.2, -0.1)	-3.0 (-3.3, -1.3)	1.9	3.3	-0.5 (-1.1, -0.2)	1.4	10
miR-486-3p	4.3 (2.5, 5.6)	4.9 (2.5, 6.2)	3.5 (3.1, 4.3)	-1.5	1.8	-2.0 (-3.1, 0.2)	**4.1**	**9**
miR-520c-3p	2.3 (0.3, 2.9)	-0.4 (-1.6, 2.4)	-1.1 (-1.4, 0.5)	6.3	10.6	0.3 (-1.7, 0.6)	-1.3	5
miR-579^c^	4.2 (3.5, 5.0)	3.5 (3.1, 3.7)	2.4 (1.8, 2.9)	1.6	3.6	-1.2 (-1.7, -0.3)	2.2	11
miR-590-3P	5.2 (2.9, 5.8)	3.2 (2.3, 3.9)	2.4 (2.2, 3.1)	3.9	6.9	-0.9 (-1.1, 0.2)	1.9	8
miR-590-5p	1.2 (0.7, 1.4)	-0.4 (-1.2, -0.2)	-1.3 (-1.6, -0.7)	3.0	5.6	-0.5 (-1.1, -0.3)	1.4	10
miR-598	3.7 (3.1, 4.3)	2.8 (2.5, 3.6)	1.5 (1.4, 1.9)	1.8	4.6	-1.2 (-1.7, -0.8)	2.2	12
miR-628-5p	3.0 (-8.4, 7.9)	-7.2 (-8.3, 3.9)	-2.9 (-7.8, 2.2)	1174.6	61.3	0.4 (-1.6, 1.7)	-1.3	5
miR-15b#	9.9 (2.6, 11.4)	6.2 (5.6, 8.4)	4.0 (2.0, 4.4)	13.3^b^	58.5^b^	-2.6 (-4.0, -1.8)	6.1^b^	11/11
miR-335#	6.1 (5.6, 8.3)	6.5 (5.2, 8.1)	4.2 (3.9, 5.1)	-1.4^b^	3.6^b^	-1.9 (-3.9, -1.0)	3.8^b^	12

microRNA (miRNA) highlighted in bold in matched samples (CCP-VERA) met criteria of median fold change (FC) ≥4 and ≥75% consistent dysregulation. If fold difference (FD) was < 1, FD = -1/FD. Estimates for each cohort were obtained at the mean age (52 years)

*dCt* delta cycle threshold, *HC* healthy controls

^a^For 3 patients, Ct values at follow up were extremely low (all ≈ 2, compared to ≈ 14 for the rest); these 3 values were considered to be inaccurate and in a conservative approach were excluded from analysis

^b^In these miRNAs, Ct was > 32 for some healthy controls and CCP+ patients at baseline. As a result, the calculated fold differences and changes may not be accurate; therefore, these genes were not deemed to have fulfilled our criteria for dysregulation, but were retained for further investigation in the validation cohort because the calculated FCs were near or above our cutoff, and all progressors showed consistent dysregulation

^c^In this miRNA mean FC was >4; miR-579 FC 4.27 (although median FC <4), furthermore consistent dysregulation was seen in 11/12 patients. As custom cards had capacity for 31 miRNAs to be evaluated these two miRNAs were selected as potentially important

^d^The original selection of miR-454 (FD 4.1 between HC-VERA) and miR-374 (FD 4.6 between HC-VERA) from the pilot phase was based on unadjusted between-group differences; following age-adjustment they no longer met our criteria

#### HC-CCP+ and HC-VERA comparison

Between the HC and CCP+ group 7 dysregulated miRNAs were identified (4 downregulated and 3 upregulated); between the HC and (matched CCP+ status to) VERA cohort, 13 dysregulated miRNAs were recorded (12 upregulated, 1 downregulated) (Table 1). As stated previously, these miRNAs, plus nine miRNAs that have been demonstrated in the literature to be involved in the pathogenesis in RA, informed the tailored array cards used for investigation of miRNA profiles from CCP+ to VERA.

#### Matched CCP+ to VERA serum miRNA evaluation

**Fig. 1:**
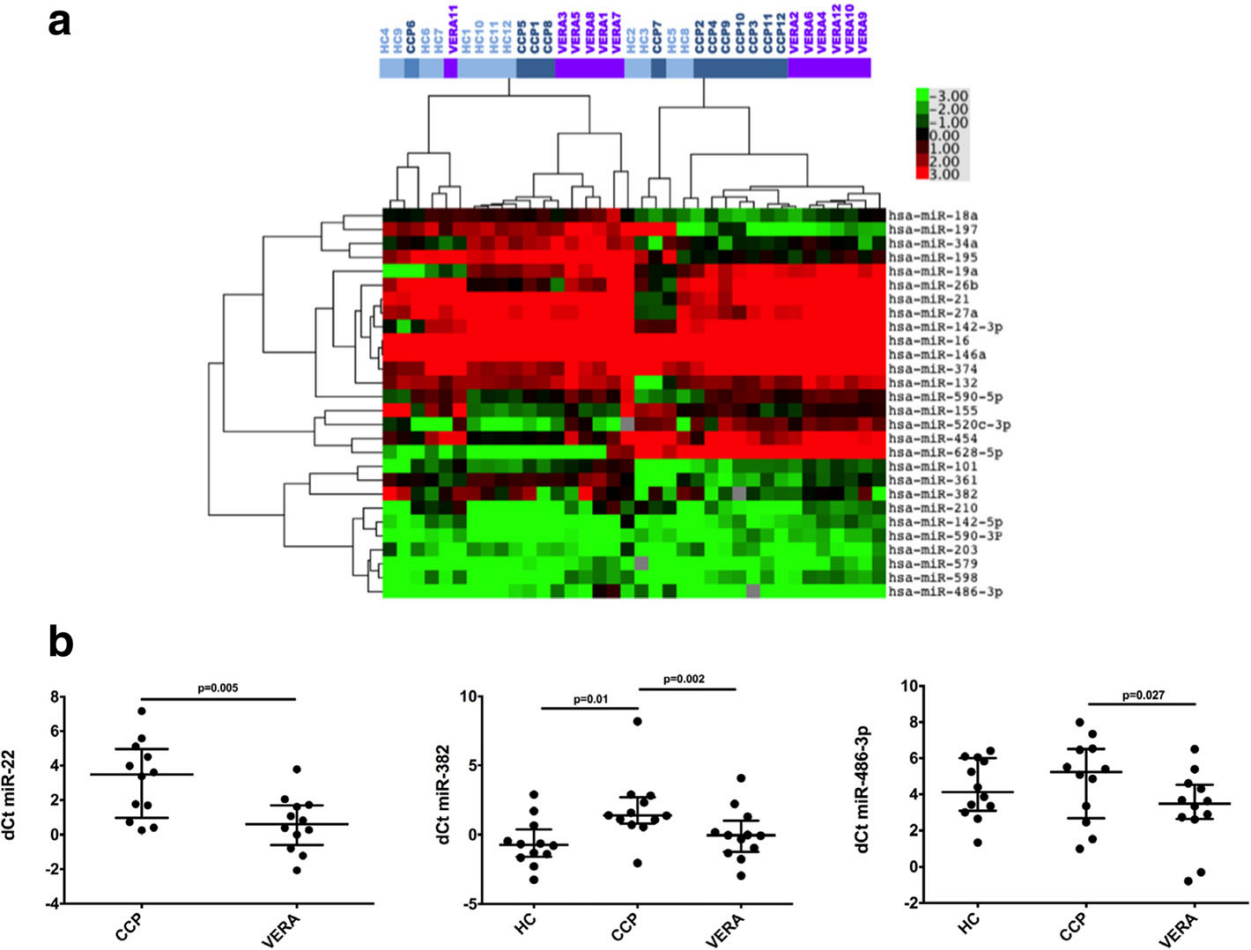
Candidate serum miRNA expression profiling. **a** MicroRNA heatmaps were generated using hierarchical clustering (Gene Cluster 3.0 and Java TreeView). Green indicates low expression; red indicates high expART ression levels. **b** Comparison of expression levels of miR-22, miR-382 and miR-486-3p in matched samples from anti-cyclic citrullinated peptide (CCP) + status to very early rheumatoid arthritis (VERA) (medians, 1^st^ to 3^rd^ quartiles). dCt*,* delta threshold cycle; HC, healthy controls. Of note, miR-22 was excluded from the healthy control cohort as it was not expressed in all 12 samples

Unsupervised hierarchical clustering of the global expression profiles of 31 miRNAs of interest was generated using complete linkage (Fig. 1a). From paired analysis of the matched samples, three circulating miRNAs were upregulated upon progression from CCP+ status to VERA (Fig. 1b). Serum miR-22 expression increased the most in patients from CCP+ status to VERA (median FC 4.3 (IQR 2.8, 12.1); expression increased in all patients). There was comparable upregulation for miR-382 (4.1 (1.7, 6.9); increased in 11/12) and miR-486-3p (4.1 (0.9, 8.6; increased in 9/12) (Table 1). Since miR-146a and miR-155 are the most commonly reported miRNAs to be dysregulated in RA, we investigated their expression in the matched serum samples. Both were upregulated in the majority (8/12) of the individuals who progressed to RA (Additional file 3); however, the FCs were not substantive (median 1.4 and 1.2 respectively).

### Validation phase of serum microRNA profiling

#### HC-CCP+ (progressor or non-progressor) and HC-VERA comparison

Between the validation HC group and the validation CCP+ progressors, two dysregulated (both upregulated) miRNAs were identified, though not entirely consistent with the pilot-phase findings, and included miR-22 with an FD of 11.3. Between the HC group and the VERA cohort, six upregulated miRNAs were identified (Additional file 4); four miRNAs were validated from the pilot results, namely miR-19a, miR-22, miR-590-3p and miR-598. The inclusion of the CCP+ non-progressor cohort provided an additional comparison to healthy status. Only miR-590-3P met the criterion of dysregulation compared to healthy status.

#### Baseline miRNA profile of progressors vs non-progressors

At baseline, the miRNAs were mostly upregulated in the progressors compared to the non-progressors, except for five miRNAs, miR-26b, miR-210, miR-486-3p, miR-590-3p and miR-628-5p (Additional file 5). Baseline FD between the two groups for the three miRNAs of interest, miR-22, miR-382 and miR-486-3p, were 19.7, 2.5 and -1.4, respectively (Table 2).

**Table 2:** Summary: within patient change for CCP+ status to VERA (progression) in both phases and CCP+ status to non-progression within patient change and vs. progressors from the validation phase

	CCP+ to VERA	CCP+ to no progression	CCP+ non progressor	CCP+ progressor	Progressors vs. non progressors			
	Median FC (IQR 1^st^, 3^rd^)	Median FC (IQR 1^st^, 3^rd^)	B/L median dCt (IQR)	B/L median dCt (IQR)	FD between medians	Area under ROC curve (90% CI)	Sens	Spec
Pilot phase								
miR-22	4.3 (2.8, 12.1)	-	-	-	-		-	-
miR-382	4.1 (1.7, 6.9)	-	-	-				
miR-486-3p	4.1 (0.9, 8.6)	-	-	-	-		-	-
Validation Phase								
miR-22	2.5 (-2.2, 15.3)	3.4 (2.3, 12.6)	7.4 (4.1, 8.2)	3.1 (1.8, 7.3)	19.7	0.68 (0.48, 0.82)	63%	100%
miR-382	1.2 (-2.1, 2.7)	2.4 (1.0, 2.6)	1.1 (0.0, 1.8)	-0.2 (-0.5, 1.9)	2.5	0.57 (0.40, 0.75)	75%	58%
miR-486-3p	2.2 (-2.5, 6.0)	1.0 (-1.4, 3.0)	3.4 (1.7, 3.9)	3.9 (2.6, 5.0)	-1.4	0.55 (0.36, 0.72)	50%	75%

*CCP* anti-cyclic citrullinated peptide, *VERA* very early rheumatoid arthritis, *miRNA* microRNA, *FC* fold change, *FD* fold difference, *B/L* baseline (sample), *IQR* interquartile range, *ROC* receiver operating characteristic, *Sens* sensitivity, *Spec* specificity

#### miRNA change in matched CCP+ to VERA progressor and matched CCP+ to non-progressor status

Unsupervised hierarchical clustering of the 31 miRNAs of interest replicating the patient groups studied in the pilot phase (HC, CCP+ and matched VERA sample) demonstrated similar clustering as in the pilot phase (Additional file 6). The 31 miRNAs did not reach our stringent pre-defined criteria. However, of the three key miRNAs identified in the pilot phase, miR-486-3p increased in progressors by a median (IQR) FC of 2.2 (0.4, 6.0) (Additional file 7, Fig. 2a) compared to stable expression within the non-progressor cohort with a FC of 1.0 (0.7, 3.0). Whilst the baseline miR-22 FD *between* progressors and non-progressors reported earlier was significant (19.7), *within* group interval change, median FC (IQR), was increased in both progressors (2.5 (0.5, 19.7)) and non-progressors (3.4 (0.5, 12.1)) (Additional file 7, Fig. 2a). Similar findings were observed with miR-382, with greater median FC (IQR) in the non-progressor cohort (2.4 (1.0, 2.6)) versus progressors (1.2 (0.5, 2.6)) (Additional file 7, Fig. 2a).

**Fig. 2:**
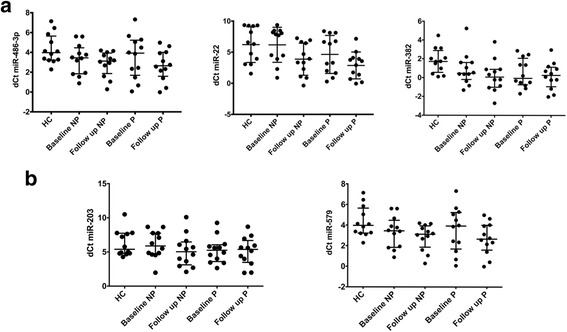
Validation-phase serum expression levels of candidate miRNAs. Baseline and follow-up relative expression in the progressor (P) and non-progressor (NP) cohorts of miR-486-3p, miR-22 and miR-382 (**a**) and miR-203 and miR-579 (**b**). HC, healthy controls; dCt, delta cycle threshold

Two miRNAs were upregulated from baseline to follow up in the non-progressor but not in the progressor group,with FC (IQR) miR-203 of 3.1 (0.5, 6.9) vs. -1.1 (0.3, 4.3) and miR-579 of 3.2 (1.0, 3.7) vs. -1.1 (0.3, 3.3), respectively (Additional file 7, Fig. 2b).

#### Association with clinical variables

The three miRNAs of interest were substantively associated (|rho| > 0.3) with the score on the patient disease activity visual analogue scale (VAS) (Additional file 8). MiR-382 and 486-3p were both also associated with tender joint count in 28 joints (TJC28) and Disease Activity Score in 28 joints based on erythrocyte sedimentation rate (DAS28-ESR).

#### Predicting progression using baseline miRNAs

Individually, miRs-197 (area under the receiver operating characteristic curve (AUROC) analysis 0.69, 90% CI 0.52, 0.85) and -335* (AUROC 0.71, 90% CI 0.52, 0.85) performed better than chance (with 90% confidence); the recorded areas under the curve (AUCs) were modest although the upper confidence intervals for several miRNAs included an AUC = 0.8 (Additional file 5). MiR-22 had sensitivity and specificity of 63% and 100%, respectively, with an AUROC of 0.68 (90% CI 0.48, 0.82), also demonstrating the highest Youden index, highlighting the importance of re-evaluating this in a larger sample size. MiR-382 and miR-486-3p performed less well, with an AUROC of 0.57 (0.40, 0.75) and 0.55 (0.36, 0.72), respectively. Table 2 summarises the within-patient change for CCP+ status to VERA (progression) in both the pilot and validation phases and within-patient change for CCP+ to non-progression and non-progression vs. progression in the validation phase.

### Pathway analysis and networking of miRNA target genes

Pathway prediction for the miRNAs of interest was performed, using a bioinformatics approach MetaCore™, to further elucidate functional processes associated with selected miRNAs and their targets. The expanded networks generated for miRNAs of interest represent predicted targets (Additional files 9, 10 and 11). Canonical interaction between the transcription factor p53 and miR-22 is highlighted; p53 plays a central role in a number of cellular functions, and is overexpressed in RA synovial tissue, and also activates miR-22 by binding to its promoter region [14, 15]. The predicted network shows that miR-486-3p has an inhibitory effect on bone morphogenetic protein 1 (BMP-1), indicative of miRNA function. MiR-382 negatively regulates the phosphatase and tensin homolog (PTEN), which is upstream of the AKT/mTOR signalling pathway.

## Discussion

This first study of miRNAs in individuals at risk of RA has identified new miRNAs of interest, which may be associated with RA initiation and progression from systemic autoimmunity to disease and may also have a predictive role in the progression from “at-risk” status to RA.

Current literature reports clinical, serological, imaging and biological markers either associated with or potentially predictive of progression from systemic autoimmunity to RA, such as ACPA, RF, and shared epitope (SE) fine mapping [16, 17]. Other biomarkers that have been explored comprise synovial tissue and histology studies, gene expression analyses and sensitive imaging [18, 19]. MiRNA studies to date have mainly focused on peripheral blood and synovial tissue expression in established RA, often in minimally defined and heterogeneous cohorts. Whilst the numbers included in our study are relatively modest, the benefit of using matched samples from well-phenotyped individuals (excuding those with ultrasound-detected synovitis, likely to have already developed inflammation on a pathophysiological level), offers a particularly robust and unique approach to identify miRNA markers of disease initiation and progression. Our study design initially considered over 700 miRNAs in the pilot phase, followed by a focused validation phase, enabling us to consider a vast number of potentially influential miRNAs.

We identified potential roles of miR-22 and -382 and confirmed the importance of miR-486-3p. Despite these miRNAs in the validation phase not meeting our stringent criteria; miR-486-3p had an FC > 2, which is of biological significance particularly since there was stable expression in the non-progressors. The validation of miR-22 upregulation in VERA and CCP states compared to health potentially implicates a role in the development of inflammatory disease. Baseline miR-22 was strongly upregulated in progressors compared to non-progressors (comparator group) and thus has potential clinical utility for identifying those that may be at greatest risk. However, the ROC analysis did not reflect this, highlighting the need for further evaluation with a larger patient cohort. Interestingly it has also been identified as a predictor of response to tumour necrosis factor-inhibitor therapy [20, 21]. The higher-than-expected FC of miR-22 and miR-382 within the non-progressors may reflect an association between this miRNA with ongoing autoimmunity, which we anticipate, with further follow up in a proportion of the non-progressors, may manifest as progression to VERA. Continued evaluation of this cohort will allow us to address this.

MiR-203 has previously been identified as an miRNA involved in RA [7], but has not been studied before in CCP+ at-risk individuals, while miR-579 was upregulated in VERA compared to HC; the significance however of upregulation in both the cohorts with inflammation and the CCP+ non-progressors remains unclear. It is acknowledged that identification of these two miRNAs is from the comparator group of non-progressors, and there has been no validation process.

MiRNA-146a and miRNA-155 were only found to be upregulated in our pilot study with progression to VERA, conceivably as different miRNAs might be implicated in autoimmunity and disease initiation compared to miRNAs in established disease. The initial studies evaluating these miRNAs at the site of disease (synovial tissue) as opposed to serum might also be relevant.

## Conclusions

In summary, we report the first study that has identified in particular three miRNAs associated with autoimmunity (at-risk RA) and the progression to RA, using a unique matched serum sample and comprehensive miRNA array approach. Given the associations between clinical markers and potential predictive ability, validation of the signature miRNAs as a next step may offer the opportunity to improve current models [5] (including evaluation in CCP+, ultrasound positive cohort), and supports investigation into the biological functions of the candidate miRNAs through future network and functional analyses.

## Additional files

Additional file 1: Baseline characteristics of individuals for pilot and validation phases. (DOCX 262 kb)

Additional file 2: Supplemental methods. (DOCX 122 kb)

Additional file 3: Serum miRNAs miR-146a and miR-155 expression levels in the pilot phase. (DOCX 19324 kb)

Additional file 4: List of miRNAs of interest with age adjustment in the validation phase. (DOCX 119 kb)

Additional file 5: Comparison of miRNA expression. (DOCX 132 kb)

Additional file 6: Heatmap for the validation phase. (DOCX 148 kb)

Additional file 7: Within-patient changes in the validation phase. (DOCX 122 kb)

Additional file 8: Associations between clinical variables and dCt at baseline for key miRNAs. (DOCX 11 kb)

Additional file 9: Network of the predicted targets of miR-22. (DOCX 425 kb)

Additional file 10: Network of the predicted targets of miR-382. (DOCX 214 kb)

Additional file 11: Network of the predicted targets of miR-486-3p. (DOCX 331 kb)

## Abbreviations

ACPA: Anti-citrullinated peptide antibodies
ACR/EULAR: American College of Rheumatology/European League Against Rheumatism
AUC: Area under the curve
AUROC: Area under the receiver operating characteristic curve
BMP-1: Bone morphogenic protein-1
CCP: Anti-cyclic citrullinated peptide
cT: Cycle threshold
dCt: Delta cycle threshold
ERA: Early rheumatoid arthritis
FC: Fold change
FD: Fold difference
HC: Healthy control
IA: Inflammatory arthritis
MCP: Metacarpophalangeal joints
miRNA: MicroRNA
MSK: Musculoskeletal
MTP: Metatarsophalangeal joints
PIP: Proximal interphalangeal joints
PTEN: Phosphatase and tensin homolog
RA: Rheumatoid arthritis
RF: Rheumatoid factor
ROC: Receiver operating characteristic
VERA: Very early rheumatoid arthritis
