# Supplementary material for: Profiling microRNAs in individuals at risk of progression to rheumatoid arthritis
Source: Arthritis Res Ther. 2017 Dec 22;19:288. doi: 10.1186/s13075-017-1492-9 (PMC5741901; doi:10.1186/s13075-017-1492-9)
Supplement: Supplementary file 1 — Baseline characteristics of individuals for pilot and validation phases. (DOCX 262 kb) [file 13075_2017_1492_MOESM1_ESM.docx]

**Additional file 1**

**Baseline characteristics of individuals for pilot and validation phases.** HC Healthy controls, IQR Interquartile range, VERA Very early rheumatoid arthritis.

| **Pilot phase** | **HC** | **CCP+ progression to VERA** | |
| --- | --- | --- | --- |
| Number | 12 | 12 | |
| Median Age (IQR) | 43 (38-55.3) | 52 (43-70) | |
| Female | 8 (67%) | 8 (67%) | |
| Median weeks to synovitis (IQR) | - | 34.5 (13.5-56.5) | |
| **Validation phase** | **HC** | **CCP+ progression to VERA** | **CCP+ no progression** |
| Number | 12 | 12 | 12 |
| Median Age (IQR) | 35.5 (29.8-43.8) | 54 (39.0-59.2) | 53.5 (50.0-65.5) |
| Female | 6 (50%) | 10 (83.3) | 6 (50) |
| Median weeks to synovitis (IQR) | - | 41 (25.8-65.0) | - |
| Median weeks follow-up (IQR) | - | - | 152 (127.3-241.5) |

**
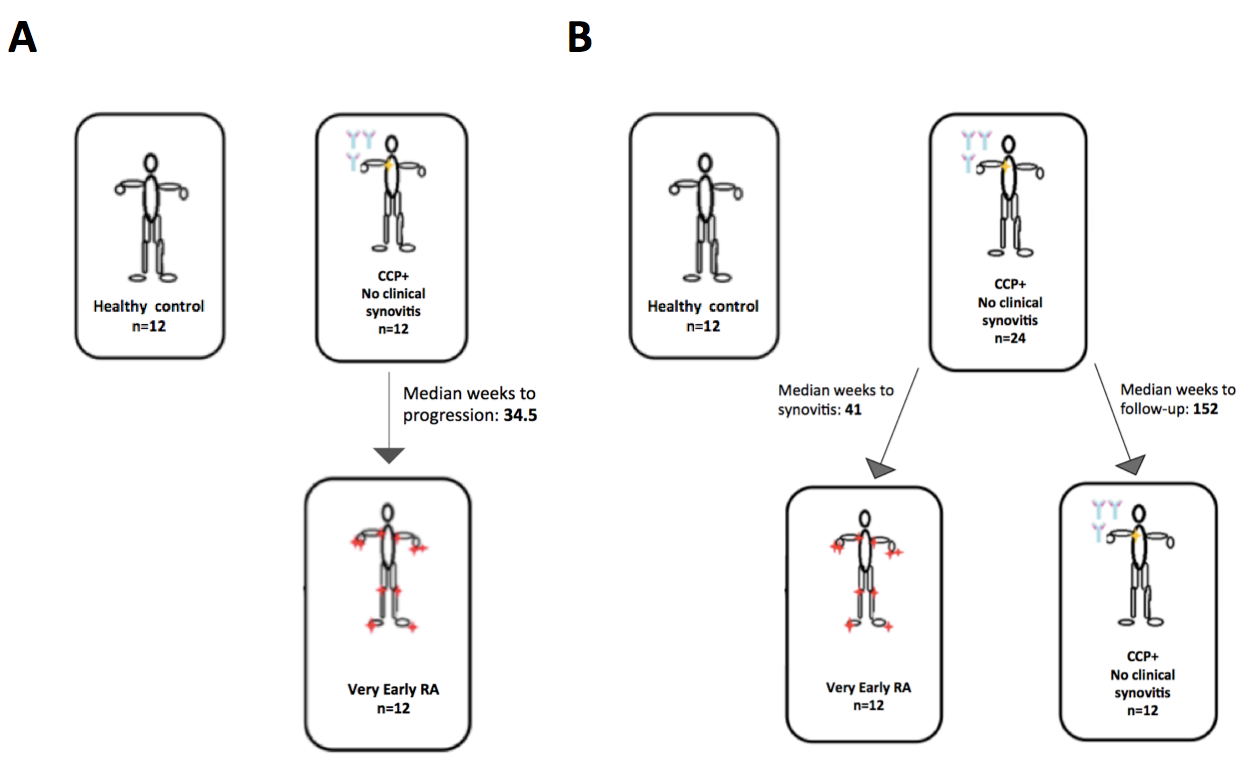
**

**(A)** Patients cohorts of serum miRNA profiling for pilot phase Healthy Controls (n=12), CCP positive (CCP) group n=12 progressed to Very Early RA (VERA) group. **(B)** Patients cohorts for validation phase including HC n=12, CCP+ progressor and 12 CCP+ non progressor groups.
