# Supplementary material for: Profiling microRNAs in individuals at risk of progression to rheumatoid arthritis
Source: Arthritis Res Ther. 2017 Dec 22;19:288. doi: 10.1186/s13075-017-1492-9 (PMC5741901; doi:10.1186/s13075-017-1492-9)
Supplement: Supplementary file 2 — Supplemental methods. (DOCX 122 kb) [file 13075_2017_1492_MOESM2_ESM.docx]

**Additional file 2**

**Supplemental methods**

*Recruitment of Health Controls*

The healthy controls were recruited through one of our observational studies in the research unit (Inflammatory Arthritis disease CONtinuum longitudinal study – IACON REC Ref: 09/H1307/98). Healthy controls attended for baseline assessment which included physical examination and a joint count. Blood was withdrawn and serum was extracted as per protocol, reflecting the same methods as in the CCP+ study. Anti-CCP and RF were performed as part of their assessments. Only individuals with a negative serology were used as healthy controls in this study. As per protocol, there was no long term follow up of healthy controls.

*Isolation and profiling of serum microRNA*

Peripheral blood samples were collected in Blood Clot activator Vacutainer (BD) tubes. Samples were left at room temperature for the blood to clot and were centrifuged at 3000rpm for 10 minutes. Serum was separated into aliquots, centrifuged once again at 13,000g for 10 minutes at 4°C. Serum collected was stored at -80°C until further use. Serum microRNAs were isolated according to the manufacturer’s protocol using miRNeasy serum plasma kit (Qiagen, UK). 1μg of MS2 RNA (Roche) and spike-in control c.elegans miR-39 mimic (3.5μl of 1.6x10^8^ copies/μl) (Qiagen) were added to denatured samples. MS2 RNA was used as an RNA stabiliser during cDNA synthesis and miR-39 enabled normalisation for any nonspecific losses incurred during miRNA purification. Final elution volume was with 20μl molecular grade water.

For cDNA synthesis, Taqman miRNA reverse transcription kit was used (Life Technologies), 3 μl RNA input isolated from serum with Megaplex primer pools Human set v3.0 A and B (Life Technologies) separately. Pre-amplification reactions were also performed following manufacturer’s protocol using Taqman pre-amplification mastermix, Taqman array human miRNA A and B (Life Technologies). The pre-amplified product can be stored at -20°C until it is run on the miRNA Taqman low density array TLDA array.

Undiluted pre-amplification product was prepared in a mastermix with Taqman universal mastermix II no UNG and water and loaded into Taqman Low density microRNA cards A v3.0 and B set v2.0 (Life Technologies) on an Applied Biosystems 7900HT fast real-time system according to the instructions provided in the manuals (Life technologies). This method uses a pre-configured micro fluidic card that enables the quantification of 754 human miRNAs. Data were automatically analysed with SDS Relative Quantification software version 2.4 (Life Technologies).

There is no current consensus of internal control for plasma miRNA. Therefore, we used the average Ct (threshold cycle) value of all miRNAs with valid amplification plots (Ct≤33). To validate the accuracy of using the average Ct value (average of total expressed miRNAs) as a reference for normalisation, and to find out the most stably expressed candidate internal controls of plasma miRNA, NormFinder software was used as described before [1], which resulted in identifying average Ct as the optimal and most stable normlisation method. The corresponding Ct value for each microRNA was therefore normalised to the average, followed by calculation of ddCt between HC-CCP, HC-VERA. Fold change (FC) represented the increase or decrease in expression based on the obtained ddCt values. For the matched samples CCP to VERA, median and mean of fold change values within the 12 samples was calculated.

*Imputation of undetermined Ct values*

In the validation phase, undetermined Ct values were imputed at the replicate level prior to analysis using R package nondetects, which employs an expectation maximisation algorithm [2]. The algorithm models the probability of an undetermined value occurring as a function of the observed values in the dataset, on the assumption that values may be undetermined due to a failure to amplify, rather than the ‘true’ value of Ct being >40.

**Supplemental references**

1. Andersen CL, Jensen JL, Ørntoft TF. Normalization of Real-Time Quantitative Reverse Transcription-PCR Data: A Model-Based Variance Estimation Approach to Identify Genes Suited for Normalization, Applied to Bladder and Colon Cancer Data Sets. *Cancer Res* 2004;64:5245-5250.
2. McCall NM, McMurray HR, Land H, Almudevar A. On non-detects in qPCR. *Bioinformatics* 2014;30:2310-2316.
