# Supplementary material for: Profiling microRNAs in individuals at risk of progression to rheumatoid arthritis
Source: Arthritis Res Ther. 2017 Dec 22;19:288. doi: 10.1186/s13075-017-1492-9 (PMC5741901; doi:10.1186/s13075-017-1492-9)
Supplement: Supplementary file 3 — Serum miRNAs miR-146a and miR-155 expression levels in the pilot phase. (DOCX 19324 kb) [file 13075_2017_1492_MOESM3_ESM.docx]

**Additional file 3**


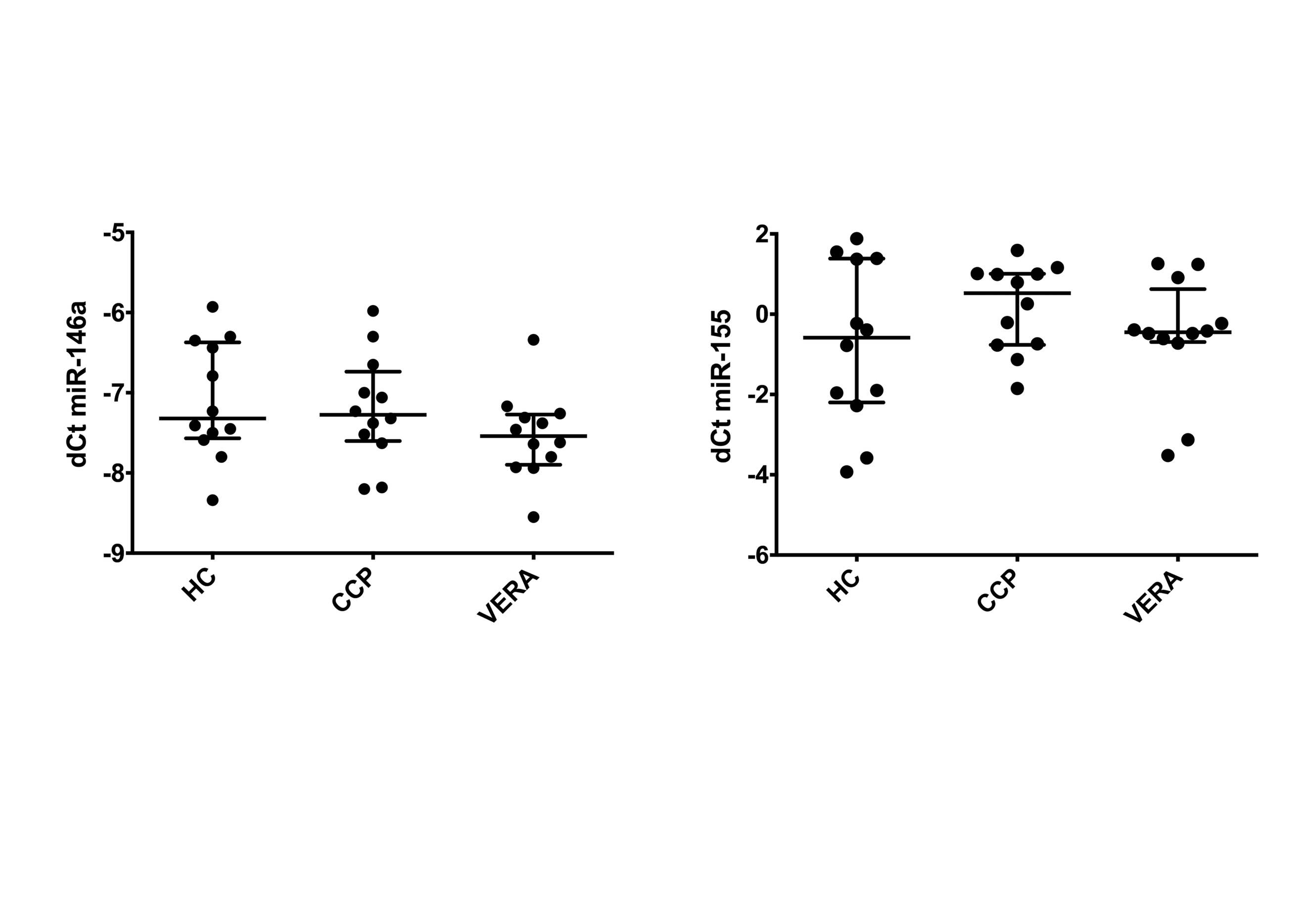


**Serum miRNAs miR-146a and miR-155 expression levels in the pilot phase.** Relative expression levels of miR-146a and miR-155 in HC, CCP and matched VERA groups [median FC 1.4 (IQR 0.8, 1.7)] and [median FC 1.2 (IQR 0.9, 2.7)] (median, 1^st^ and 3^rd^ quartiles represented)
