# Supplementary material for: Profiling microRNAs in individuals at risk of progression to rheumatoid arthritis
Source: Arthritis Res Ther. 2017 Dec 22;19:288. doi: 10.1186/s13075-017-1492-9 (PMC5741901; doi:10.1186/s13075-017-1492-9)
Supplement: Supplementary file 4 — List of miRNAs of interest with age adjustment in the validation phase. (DOCX 119 kb) [file 13075_2017_1492_MOESM4_ESM.docx]

**Additional file 4**

**List of miRNAs of interest with age-adjusted FD≥4 between the three studied cohorts in the validation phase** (🡹upregulated FC≥4 in both phases, 🡻downregulated FC≤-4 in both phase). MiRNA highlighted in bold in matched samples (CCP-VERA) met criteria of median FC≥4 and ≥75% consistent dysregulation in the pilot phase. FD=fold difference; FC=fold change. If FD<1, FD=-1/FD. Estimates for each cohort were obtained at the mean age (48 years).

|  | HC | CCP (non-prog) | CCP (prog) | VERA | CCP (non-prog) vs. HC | CCP (prog) vs. HC | VERA vs. HC |
| --- | --- | --- | --- | --- | --- | --- | --- |
| miR | dCt median (IQR) | dCt median (IQR) | dCt median (IQR) | dCt median (IQR) | FD between medians | FD between medians | FD between medians |
| miR-16 | -7.9 (-8.8, -6.8) | -8.2 (-8.8, -7.2) | -8.9 (-10.5, -7.8) | -9.5 (-11.3, -8.0) | 1.2 | 2.0 | 3.0 |
| miR-18a | 0.1 (-0.6, 0.6) | -1.1 (-1.3, -0.7) | -1.1 (-2.8, -0.5) | -1.9 (-3.0, -0.4) | 2.3 | 2.3 | 4.0 |
| miR-19a | -0.8 (-0.9, -0.4) | -1.7 (-2.3, -1.4) | -1.9 (-3.3, -1.0) | -2.8 (-3.6, -1.2) | 1.9 | 2.1 | 4.0 (🡹) |
| miR-21 | -2.1 (-2.4, -1.6) | -2.6 (-3.4, -2.4) | -3.7 (-4.9, -3.0) | -4.3 (-5.3, -1.5) | 1.4 | 3.0 | 4.6 |
| **miR-22** | **6.6 (3.8, 9.0)** | **7.4 (4.1, 8.2)** | **3.1 (1.8, 7.3)** | **3.3 (1.0, 4.3)** | **-1.7** | **11.3** | **9.8 (**🡹**)** |
| miR-26b | -0.4 (-1.0, -0.2) | -1.5 (-1.9, -1.0) | -1.3 (-3.2, -0.7) | -1.8 (-3.4, -0.7) | 2.1 | 1.9 | 2.6 |
| miR-34a | 3.4 (2.4, 4.8) | 3.0 (1.7, 4.0) | 1.4 (0.7, 3.2) | 2.5 (1.0, 3.1) | 1.3 | 4.0 | 1.9 |
| miR-101 | 2.3 (2.3, 3.7) | 3.2 (2.6, 3.7) | 2.1 (0.5, 3.3) | 1.6 (0.2, 3.3) | -1.9 | 1.1 | 1.6 |
| miR-132 | 0.8 (-0.2, 1.4) | 0.2 (-0.6, 1.3) | -0.2 (-1.9, 0.1) | -0.7 (-1.7, 0.3) | 1.5 | 2.0 | 2.8 |
| miR-142-3p | -3.8 (-3.7, -3.0) | -3.9 (-5.1, -3.5) | -4.4 (-5.2, -3.7) | -4.4 (-5.2, -2.6) | 1.1 | 1.5 | 1.5 |
| miR-142-5p | 2.0 (1.9, 2.5) | 1.7 (0.8, 1.9) | 1.4 (-0.1, 2.3) | 0.6 (-0.4, 2.5) | 1.2 | 1.5 | 2.6 |
| miR-146a | -6.2 (-6.9, -4.7) | -6.1 (-7.3, -5.3) | -7.3 (-8.3, -6.4) | -7.1 (-8.3, -6.6) | -1.1 | 2.1 | 1.9 |
| miR-155 | -0.4 (-1.0, 0.4) | -0.8 (-1.7, 0.3) | -1.3 (-1.8, -0.4) | -1.3 (-1.9, -1.1) | 1.3 | 1.9 | 1.9 |
| miR-195 | -1.4 (-2.0, -0.9) | -1.9 (-2.4, -0.7) | -2.9 (-3.6, -1.4) | -3.0 (-3.5, -1.7) | 1.4 | 2.8 | 3.0 |
| miR-197 | -2.2 (-2.8, -1.1) | -2.6 (-3.6, -1.7) | -4.0 (-4.2, -2.5) | -3.3 (-3.9, -2.9) | 1.3 | 3.5 | 2.1 |
| miR-203 | 5.3 (4.8, 7.8) | 6.0 (4.6, 7.6) | 5.2 (3.5, 6.0) | 5.2 (3.9, 5.9) | -1.6 | 1.1 | 1.1 |
| miR-210 | 1.9 (1.3, 2.6) | 0.9 (0.3, 1.7) | 1.3 (-0.5, 1.7) | 0.1 (-0.7, 2.2) | 2.0 | 1.5 | 3.5 |
| miR-223 | -10.7 (-11.1, -10.4) | -10.7 (-12.0, -9.9) | -11.5 (-12.4, -11.1) | -12.2 (-13.2, -10.9) | 1.0 | 1.7 | 2.8 |
| miR-361 | 2.4 (1.6, 3.3) | 3.3 (1.8, 3.5) | 1.6 (0.5, 2.5) | 1.7 (0.5, 2.3) | -1.9 | 1.7 | 1.6 |
| miR-374 | 0.1 (-0.3, 0.3) | -0.7 (-1.3, 0.1) | -0.8 (-1.3, -0.4) | -0.8 (-2.5, 0.3) | 1.7 | 1.9 | 1.9 |
| **miR-382** | **1.3 (0.9, 2.2)** | **1.1 (0.0, 1.8)** | **-0.2 (-0.5, 1.9)** | **0.5 (-0.2, 1.0)** | **1.1** | **2.8** | **1.7** |
| miR-454 | -1.3 (-1.6, -0.7) | -2.0 (-2.6, -1.7) | -2.0 (-2.6, -1.1) | -2.2 (-3.1, -1.1) | 1.6 | 1.6 | 1.9 |
| **miR-486-3p** | **4.0 (2.8, 5.1)** | **3.4 (1.7, 3.9)** | **3.9 (2.6, 5.0)** | **2.6 (1.9, 4.0)** | **1.5** | **1.1** | **2.6** |
| miR-520c-3p | -2.5 (-3.3, -0.8) | -2.2 (-4.1, -2.1) | -2.9 (-4.8, -2.7) | -3.1 (-4.4, -2.3) | -1.2 | 1.3 | 1.5 |
| miR-579 | 5.1 (4.2, 5.9) | 5.4 (4.4, 6.1) | 3.9 (2.2, 5.9) | 3.9 (2.4, 4.7) | -1.2 | 2.3 | 2.3 |
| miR-590-3P | 7.9 (6.7, 7.4) | 5.4 (4.1, 7.7) | 6.3 (5.1, 8.4) | 5.9 (5.4, 7.7) | 5.7 | 3.0 | 4.0 (🡹) |
| miR-590-5p | 2.8 (2.7, 3.2) | 2.6 (1.7, 3.8) | 1.9 (1.3, 3.1) | 2.3 (0.7, 4.3) | 1.1 | 1.9 | 1.4 |
| miR-598 | 3.2 (2.2, 3.7) | 2.1 (1.4, 3.3) | 1.6 (0.9, 2.2) | 1.1 (0.0, 2.7) | 2.1 | 3.0 | 4.3 (🡹) |
| miR-628-5p | 4.1 (3.3, 5.3) | 3.4 (2.4, 3.6) | 3.5 (2.7, 4.4) | 2.6 (2.1, 3.3) | 1.6 | 1.5 | 2.8 |
| miR-15b# | 1.5 (1.0, 1.5) | 0.7 (0.4, 1.3) | 0.5 (-0.1, 1.6) | 0.2 (-1.0, 0.6) | 1.7 | 2.0 | 2.5 |
| miR-335# | 5.3 (4.2, 6.6) | 4.2 (2.9, 5.4) | 2.8 (2.2, 3.7) | 2.2 (1.6, 4.4) | 2.1 | 5.7 | 8.6 |
