# Supplementary material for: Profiling microRNAs in individuals at risk of progression to rheumatoid arthritis
Source: Arthritis Res Ther. 2017 Dec 22;19:288. doi: 10.1186/s13075-017-1492-9 (PMC5741901; doi:10.1186/s13075-017-1492-9)
Supplement: Supplementary file 5 — Comparison of miRNA expression. (DOCX 132 kb) [file 13075_2017_1492_MOESM5_ESM.docx]

**Additional file 5**

**Comparison of miRNA expression between baseline samples within the CCP+ progressors and CCP+ non-progressors cohorts.** miRs highlighted in bold satisfied criteria for dysregulation in the pilot phase. Estimates of dCt and FD for each cohort were obtained at the mean age (48 years).

| miRNA | CCP+ non progressors | CCP+ progressors | Progressors vs. non progressors | | | | |
| --- | --- | --- | --- | --- | --- | --- | --- |
|  | B/L median dCt (IQR) | B/L median dCt (IQR) | FD between medians | Area under ROC curve (90% CI) | Sensitivity* | Specificity* | Youden J |
| miR-16 | -8.2 (-8.8, -7.2) | -8.9 (-10.5, -7.8) | 1.6 | 0.59 (0.40, 0.75) | 33% | 92% | 0.25 |
| miR-18a | -1.1 (-1.3, -0.7) | -1.1 (-2.8, -0.5) | 1.0 | 0.55 (0.36, 0.72) | 83% | 42% | 0.25 |
| miR-19a | -1.7 (-2.3, -1.4) | -1.9 (-3.3, -1.0) | 1.1 | 0.53 (0.36, 0.72) | 83% | 42% | 0.25 |
| miR-21 | -2.6 (-3.4, -2.4) | -3.7 (-4.9, -3.0) | 2.1 | 0.65 (0.44, 0.79) | 50% | 83% | 0.33 |
| **miR-22** | **7.4 (4.1, 8.2)** | **3.1 (1.8, 7.3)** | **19.7†** | **0.68 (0.48, 0.82**) | **63%** | **100%** | **0.63** |
| miR-26b | -1.5 (-1.9, -1.0) | -1.3 (-3.2, -0.7) | -1.1 | 0.53 (0.36, 0.72) | 83% | 50% | 0.33 |
| miR-34a | 3.0 (1.7, 4.0) | 1.4 (0.7, 3.2) | 3.0 | 0.63 (0.44, 0.79) | 75% | 58% | 0.33 |
| miR-101 | 3.2 (2.6, 3.7) | 2.1 (0.5, 3.3) | 2.1 | 0.64 (0.44, 0.79) | 83% | 67% | 0.50 |
| miR-132 | 0.2 (-0.6, 1.3) | -0.2 (-1.9, 0.1) | 1.3 | 0.66 (0.48, 0.82) | 50% | 92% | 0.42 |
| miR-142-3p | -3.9 (-5.1, -3.5) | -4.4 (-5.2, -3.7) | 1.4 | 0.53 (0.36, 0.72) | 42% | 75% | 0.17 |
| miR-142-5p | 1.7 (0.8, 1.9) | 1.4 (-0.1, 2.3) | 1.2 | 0.58 (0.40, 0.75) | 67% | 67% | 0.33 |
| miR-146a | -6.1 (-7.3, -5.3) | -7.3 (-8.3, -6.4) | 2.3 | 0.65 (0.48, 0.82) | 42% | 92% | 0.33 |
| miR-155 | -0.8 (-1.7, 0.3) | -1.3 (-1.8, -0.4) | 1.4 | 0.63 (0.44, 0.79) | 75% | 58% | 0.33 |
| miR-195 | -1.9 (-2.4, -0.7) | -2.9 (-3.6, -1.4) | 2.0 | 0.63 (0.44, 0.79) | 83% | 50% | 0.33 |
| miR-197 | -2.6 (-3.6, -1.7) | -4.0 (-4.2, -2.5) | 2.6 | 0.69 (0.52, 0.85) | 92% | 58% | 0.50 |
| miR-203 | 6.0 (4.6, 7.6) | 5.2 (3.5, 6.0) | 1.7**†** | 0.60 (0.44, 0.79) | 50% | 83% | 0.33 |
| miR-210 | 0.9 (0.3, 1.7) | 1.3 (-0.5, 1.7) | -1.3 | 0.57 (0.40, 0.75) | 92% | 42% | 0.33 |
| miR-223 | -10.7 (-12.0, -9.9) | -11.5 (-12.4, -11.1) | 1.7 | 0.60 (0.40, 0.75) | 33% | 92% | 0.25 |
| miR-361 | 3.3 (1.8, 3.5) | 1.6 (0.5, 2.5) | 3.2 | 0.67 (0.48, 0.82) | 67% | 75% | 0.42 |
| miR-374 | -0.7 (-1.3, 0.1) | -0.8 (-1.3, -0.4) | 1.1 | 0.51 (0.32, 0.68) | 17% | 92% | 0.08 |
| **miR-382** | **1.1 (0.0, 1.8)** | **-0.2 (-0.5, 1.9)** | **2.5** | **0.57 (0.40, 0.75)** | **75%** | **58%** | **0.33** |
| miR-454 | -2.0 (-2.6, -1.7) | -2.0 (-2.6, -1.1) | 1.0 | 0.53 (0.36, 0.72) | 50% | 67% | 0.17 |
| **miR-486-3p** | **3.4 (1.7, 3.9)** | **3.9 (2.6, 5.0)** | **-1.4** | **0.55 (0.36, 0.72)** | **50%** | **75%** | **0.25** |
| miR-520c-3p | -2.2 (-4.1, -2.1) | -2.9 (-4.8, -2.7) | 1.6 | 0.64 (0.44, 0.79) | 58% | 83% | 0.42 |
| miR-579 | 5.4 (4.4, 6.1) | 3.9 (2.2, 5.9) | 2.8 | 0.68 (0.48, 0.82) | 83% | 67% | 0.25 |
| miR-590-3P | 5.4 (4.1, 7.7) | 6.3 (5.1, 8.4) | -1.9**†** | 0.54 (0.36, 0.72) | 75% | 50% | 0.25 |
| miR-590-5p | 2.6 (1.7, 3.8) | 1.9 (1.3, 3.1) | 1.6 | 0.58 (0.40, 0.75) | 83% | 42% | 0.25 |
| miR-598 | 2.1 (1.4, 3.3) | 1.6 (0.9, 2.2) | 1.4 | 0.62 (0.44, 0.79) | 33% | 92% | 0.25 |
| miR-628-5p | 3.4 (2.4, 3.6) | 3.5 (2.7, 4.4) | -1.1**†** | 0.55 (0.36, 0.72) | 17% | 100% | 0.17 |
| miR-15b#* | 0.7 (0.4, 1.3) | 0.5 (-0.1, 1.6) | 1.1 | 0.58 (0.40, 0.75) | 83% | 50% | 0.33 |
| miR-335#* | 4.2 (2.9, 5.4) | 2.8 (2.2, 3.7) | 2.6**†** | 0.71 (0.52, 0.85) | 50% | 92% | 0.42 |
