# Supplementary material for: Profiling microRNAs in individuals at risk of progression to rheumatoid arthritis
Source: Arthritis Res Ther. 2017 Dec 22;19:288. doi: 10.1186/s13075-017-1492-9 (PMC5741901; doi:10.1186/s13075-017-1492-9)
Supplement: Supplementary file 6 — Heatmap for the validation phase. (DOCX 148 kb) [file 13075_2017_1492_MOESM6_ESM.docx]

**Additional file 6**


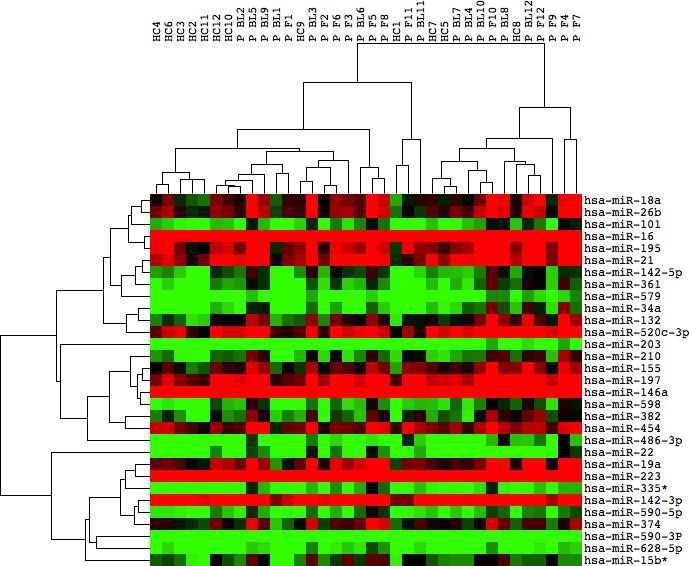


**Heatmap for validation phase**. For healthy cohort (HC) and progressors baseline (P BL) and follow up samples (P F). MicroRNAs heatmaps were generated using hierarchical clustering (Gene Cluster 3.0 and Java TreeView) *Green* indicates low expression; *Red* indicates high expression levels.
