# Supplementary material for: Profiling microRNAs in individuals at risk of progression to rheumatoid arthritis
Source: Arthritis Res Ther. 2017 Dec 22;19:288. doi: 10.1186/s13075-017-1492-9 (PMC5741901; doi:10.1186/s13075-017-1492-9)
Supplement: Supplementary file 7 — Within-patient changes in the validation phase. (DOCX 122 kb) [file 13075_2017_1492_MOESM7_ESM.docx]

**Additional file 7**

**Within-patient changes in 31 miRNAs of interest** **for CCP+ patients in the validation phase**. MiRNA highlighted in bold met criteria of median FC≥4 and ≥75% consistent dysregulation in the pilot phase.

|  |  | Validation phase | | | | | | Pilot phase from Table 1 | |
| --- | --- | --- | --- | --- | --- | --- | --- | --- | --- |
|  |  | CCP+ non progressors | | | CCP+ progressors | | | CCP+ progressors | |
|  | miR | Median (IQR) ddCt,  mean (SD) | FC | N upregulated (/12) | Median (IQR) ddCt,  mean (SD) | FC | N upregulated (/12) | Median FC | N upregulated (/12) |
| 1 | miR-16 | -0.3 (-1.5, 0.3), -0.7 (1.4) | 1.2 | 8 | -0.9 (-2.1, 1.3), -0.7 (2.3) | 1.9 | 7 | 1.3 | 10 |
| 2 | miR-18a | -0.1 (-1.0, 0.1), -0.5 (1.3) | 1.1 | 8 | -0.1 (-1.8, 0.7), -0.4 (1.7) | 1.1 | 6 | 3.1 | 10 |
| 3 | miR-19a | -0.4 (-1.4, 0.2), -0.7 (1.3) | 1.3 | 7 | -0.2 (-1.9, 0.6), -0.5 (1.8) | 1.2 | 7 | 1.5 | 9 |
| 4 | miR-21 | -0.5 (-1.3, 0.5), -0.5 (1.4) | 1.4 | 8 | 0.0 (-1.4, 1.1), 0.0 (1.5) | 1.0 | 6 | 1.6 | 9 |
| 5 | **miR-22** | **-1.8 (-3.6, 1.1), -2.3 (1.7)** | **3.4**^†^ | **12** | **-1.3 (-4.3, 1.1), -1.8 (3.6)** | **2.5**^†^ | **8** | **4.3** | **12** |
| 6 | miR-26b | -0.3 (-1.1, 0.4), -0.3 (1.3) | 1.3 | 7 | -0.4 (-1.9, 0.4), -0.5 (1.6) | 1.3 | 7 | 1.7 | 10 |
| 7 | miR-34a | -0.4 (-1.7, 0.9), -0.4 (1.8) | 1.3 | 7 | 0.3 (-1.9, 1.6), -0.1 (1.9) | -1.2 | 6 | 1.1 | 6 |
| 8 | miR-101 | -0.4 (-1.8, 0.1), -0.8 (1.3) | 1.3 | 8 | -0.1 (-1.8, 1.0), -0.3 (1.8) | 1.1 | 7 | 2.1 | 11 |
| 9 | miR-132 | -0.5 (-1.2, 0.3), -0.4 (1.3) | 1.4 | 8 | 0.3 (-1.7, 1.4), -0.2 (1.8) | -1.2 | 5 | 1.7 | 11 |
| 10 | miR-142-3p | -0.1 (-1.3, 0.6), -0.2 (1.5) | 1.1 | 7 | -0.1 (-1.1, 1.2), 0.2 (1.4) | 1.1 | 6 | 1.4 | 10 |
| 11 | miR-142-5p | -0.6 (-1.2, 0.3), -0.4 (1.3) | 1.5 | 7 | -0.1 (-1.3, 0.7), -0.3 (1.3) | 1.1 | 6 | 2.4 | 10 |
| 12 | miR-146a | -0.5 (-1.3, 0.0), -0.5 (1.4) | 1.4 | 9 | 0.1 (-1.0, 1.1), -0.2 (1.5) | -1.1 | 6 | 1.4 | 8 |
| 13 | miR-155 | -0.5 (-1.2, 0.4), -0.3 (1.2) | 1.4 | 8 | 0.1 (-1.3, 0.9), -0.2 (1.4) | -1.1 | 6 | 1.2 | 8 |
| 14 | miR-195 | -0.2 (-1.4, 0.4), -0.6 (1.4) | 1.2 | 7 | -0.1 (-2.0, 1.4), -0.5 (2.2) | 1.0 | 6 | 2.1 | 11 |
| 15 | miR-197 | -0.6 (-1.3, 0.2), -0.5 (1.2) | 1.5 | 8 | 0.5 (-1.1, 0.9), -0.1 (1.5) | -1.5 | 5 | 1.5 | 7 |
| 16 | miR-203 | -1.6 (-2.8, 0.9), -0.9 (2.8) | 3.1^†^ | 9 | 0.1 (-2.1, 1.8), -0.2 (2.6) | -1.1^†^ | 5 | 1.0 | 6 |
| 17 | miR-210 | -0.4 (-1.2, 0.1), -0.5 (1.2) | 1.3 | 8 | -0.2 (-1.9, 1.7), -0.1 (2.1) | 1.2 | 7 | 3.4 | 10 |
| 18 | miR-223 | -0.7 (-1.6, 0.4), -0.5 (1.5) | 1.6 | 8 | -0.3 (-1.1, 0.4), -0.4 (1.2) | 1.3 | 8 | 1.3 | 6/9 |
| 19 | miR-361 | -0.6 (-1.5, 0.5), -0.4 (1.3) | 1.5 | 8 | 0.3 (-1.2, 0.9), -0.2 (1.6) | -1.2 | 5 | 2.9 | 11 |
| 20 | miR-374 | -0.5 (-0.7, 0.3), -0.3 (1.3) | 1.4 | 7 | -0.3 (-1.3, 0.6), -0.2 (1.5) | 1.2 | 6 | 1.5 | 12 |
| 21 | **miR-382** | **-1.3 (-1.4, 0.0), -0.7 (1.4)** | **2.4** | **9** | **-0.2 (-1.4, 1.1), -0.3 (1.6)** | **1.2** | **6** | **4.1** | **11** |
| 22 | miR-454 | -0.1 (-0.9, 0.3), -0.2 (1.1) | 1.0 | 7 | -0.5 (-1.8, 1.1), -0.3 (1.6) | 1.4 | 8 | 1.4 | 10 |
| 23 | **miR-486-3p** | **0.0 (-1.6, 0.5), -0.5 (1.4)** | **1.0** | **6** | **-1.1 (-2.6, 1.3), -0.9 (2.8)** | **2.2** | **7** | **4.1** | **9** |
| 24 | miR-520c-3p | -0.8 (-1.4, 0.2), -0.4 (1.6) | 1.7 | 9 | 0.3 (-1.1, 2.1), 0.4 (1.9) | -1.2 | 4 | -1.3 | 5 |
| 25 | miR-579 | -1.7 (-1.9, 0.0), -0.8 (2.0) | 3.2^†^ | 9 | 0.1 (-1.7, 1.6), -0.5 (2.5) | -1.1 | 5 | 2.2 | 11 |
| 26 | miR-590-3P | -0.4 (-1.2, 1.9), 0.0 (2.1) | 1.3^†^ | 6 | -0.5 (-0.9, 1.3), -0.2 (1.8) | 1.4^†^ | 7 | 1.9 | 8 |
| 27 | miR-590-5p | -0.5 (-1.2, 0.5), -0.5 (1.4) | 1.4 | 8 | -0.4 (-1.3, 1.6), 0.1 (1.5) | 1.3 | 7 | 1.4 | 10 |
| 28 | miR-598 | -0.5 (-1.0, 0.0), -0.5 (1.1) | 1.4 | 9 | 0.2 (-1.2, 1.3), 0.0 (1.6) | -1.1 | 5 | 2.2 | 12 |
| 29 | miR-628-5p | -0.3 (-0.8, 1.1), 0.1 (1.9) | 1.2^†^ | 7 | -1.0 (-1.9, 0.6), -0.8 (1.6) | 2.0^†^ | 7 | -1.3 | 5 |
| 30 | miR-15b | -0.6 (-1.4, 0.0), -0.6 (1.3) | 1.5 | 9 | -0.1 (-1.2, 0.2), -0.3 (1.5) | 1.1 | 7 | 6.1^†^ | 11/11 |
| 31 | miR-335 | -0.2 (-1.6, 0.7), -0.5 (1.6) | 1.2^†^ | 8 | -0.7 (-1.7, 0.6), -0.5 (1.7) | 1.7 | 9 | 3.8^†^ | 12 |
