# Supplementary material for: Profiling microRNAs in individuals at risk of progression to rheumatoid arthritis
Source: Arthritis Res Ther. 2017 Dec 22;19:288. doi: 10.1186/s13075-017-1492-9 (PMC5741901; doi:10.1186/s13075-017-1492-9)
Supplement: Supplementary file 8 — Associations between clinical variables and dCt at baseline for key miRNAs. (DOCX 11 kb) [file 13075_2017_1492_MOESM8_ESM.docx]

**Additonal file 8**

**Associations between clinical variables and dCt at baseline for key miRs** (pooled progressors and non-progressors n=24).

| Baseline variable | miR-22 | miR-382 | miR-486-3P |
| --- | --- | --- | --- |
| Age | 0.31 | 0.01 | -0.10 |
| EMS | -0.19 | 0.07 | -0.10 |
| TJC28 | -0.23 | -0.46 | -0.39 |
| Patient DA VAS^1^ | -0.33 | -0.49 | -0.65 |
| Physician DA VAS^2^ | 0.24 | -0.31 | -0.28 |
| hsCRP^3^ | 0.30 | -0.03 | 0.01 |
| ESR^4^ | 0.05 | -0.12 | -0.19 |
| DAS28ESR^5^ | -0.08 | -0.40 | -0.40 |

Values presented are Spearman’s rho unless otherwise stated.

^1^n=20, ^2^n=16, ^3^n=19, ^4^n=22, ^5^n=18

*EMS early morning stiffness, TJC28 tender joint count 28, DA VAS disease activity visual analogue score, CRP C-reactive protein, ESR erythrocyte sedimentation rate, DAS28ESR disease activity score using 28 joints and ESR*
