# Supplementary material for: Profiling microRNAs in individuals at risk of progression to rheumatoid arthritis
Source: Arthritis Res Ther. 2017 Dec 22;19:288. doi: 10.1186/s13075-017-1492-9 (PMC5741901; doi:10.1186/s13075-017-1492-9)
Supplement: Supplementary file 10 — Network of the predicted targets of miR-382. (DOCX 214 kb) [file 13075_2017_1492_MOESM10_ESM.docx]

**Additional file 10**


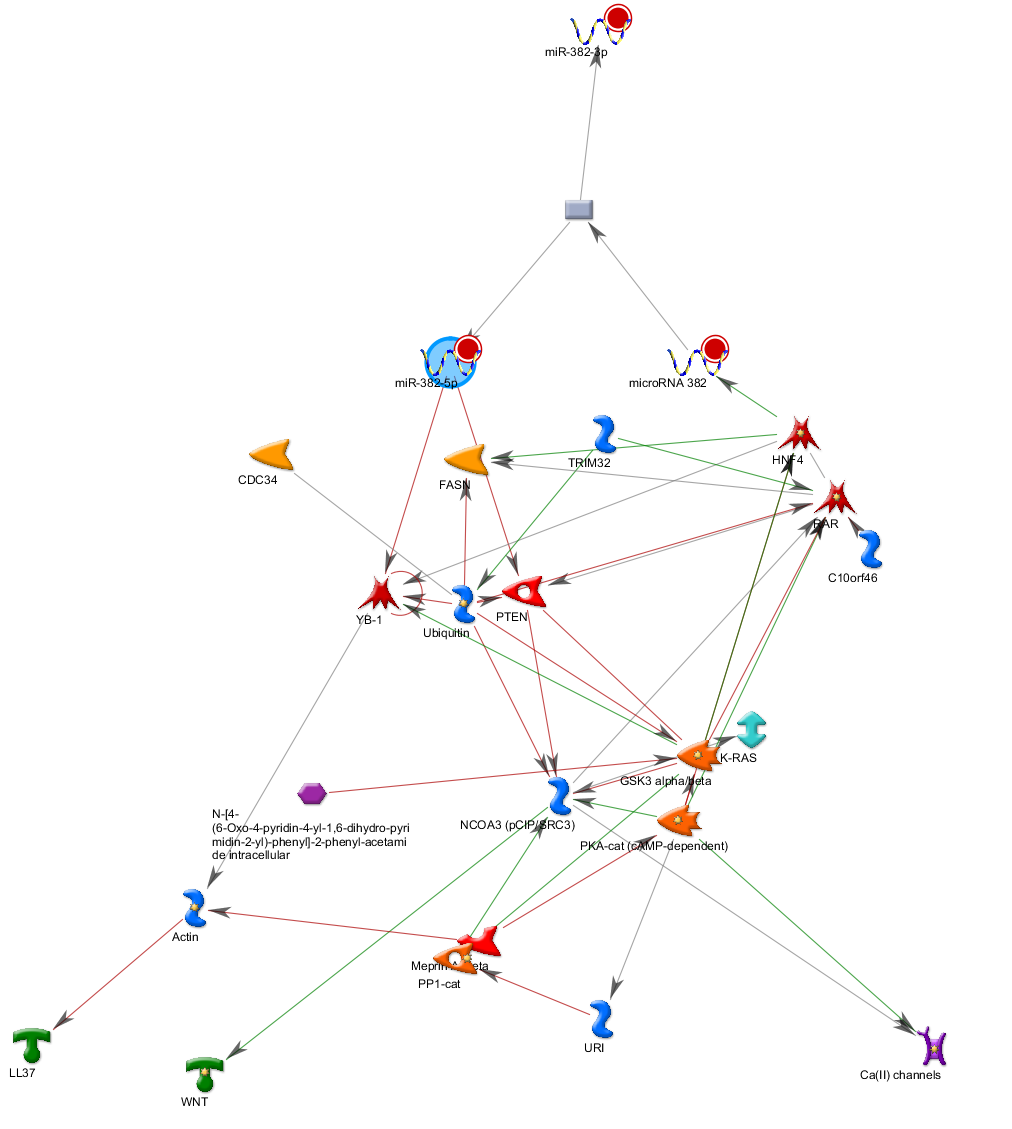


**Network of the predicted targets of miR-382 predicted target expanded network.**

Red line is indicative of a negative effect (inhibition) and green line a positive effect (activation).
